# Lessons from SARS-CoV-2 Pandemics: How Restrictive Measures Impacted the Trend of Respiratory Infections in Neonates and Infants up to Three Months of Age

**DOI:** 10.3390/pathogens11101086

**Published:** 2022-09-23

**Authors:** Domenico Umberto De Rose, Stefano Caoci, Cinzia Auriti, Chiara Maddaloni, Irma Capolupo, Guglielmo Salvatori, Carla Brusco, Luana Coltella, Carlo Concato, Cristina Russo, Luna Colagrossi, Carlo Federico Perno, Annabella Braguglia, Alberto Villani, Andrea Dotta, Massimiliano Raponi

**Affiliations:** 1 Neonatal Intensive Care Unit, Medical and Surgical Department of Fetus, Newborn and Infant, “Bambino Gesù” Children’s Hospital IRCCS, 00165 Rome, Italy; 2 Medical Direction, “Bambino Gesù” Children’s Hospital IRCCS, 00165 Rome, Italy; 3 Microbiology and Diagnostic Immunology Unit, Department of Diagnostic and Laboratory Medicine, “Bambino Gesù” Children’s Hospital IRCCS, 00165 Rome, Italy; 4 Neonatal Sub-Intensive Care Unit and Follow-Up, Medical and Surgical Department of Fetus, Newborn and Infant, “Bambino Gesù” Children’s Hospital IRCCS, 00165 Rome, Italy; 5 General Pediatric Unit, Pediatric Emergency and General Pediatric Department, “Bambino Gesù” Children’s Hospital IRCCS, 00165 Rome, Italy

**Keywords:** bronchiolitis, newborns, infants, SARS-CoV-2, COVID-19, RSV, rhinovirus, coronavirus, influenza

## Abstract

(1) Background: Massive social efforts to prevent the spread of the severe acute respiratory syndrome coronavirus 2 (SARS-CoV-2) pandemic have affected the epidemiological features of respiratory infections. (2) Methods: The study aims to describe the trend of hospitalizations for bronchiolitis among newborns and infants up to three months of life in Rome (Italy), in the pre-COVID-19 era and during the pandemic. (3) Results: We observed a marked decrease in the number of neonates and infants with bronchiolitis after national lockdowns in 2020 and the first months of 2021 and a similar trend in the number of bronchiolitis caused by respiratory syncytial virus (RSV). RSV was the leading pathogen responsible for bronchiolitis before the national lockdown in March 2020 (70.0% of cases), while Rhinovirus was the leading pathogen responsible for bronchiolitis (62.5%) during the pandemic while strict restrictions were ongoing. As Italy approached the COVID-19 vaccination target, the national government lifted some COVID-19-related restrictions. A surprising rebound of bronchiolitis (particularly cases caused by RSV) was observed in October 2021. (4) Conclusions: In this study, we describe for the first time the fluctuations over time of RSV bronchiolitis among newborns and young infants in Italy in relation to the restrictive measures containing the spread of the COVID-19 pandemic. Our results are in line with other countries’ reports.

## 1. Introduction

Italy was the first European country to announce severe nationwide restrictions in March 2020 to prevent the spread of severe acute respiratory syndrome coronavirus 2 (SARS-CoV-2) after the first clusters in the Northern regions. The response to the pandemic caused many important changes in daily lifestyle, with the activation of social measures of distancing, teleworking, closing of schools and kindergartens, closing of commercial activities, strict hygiene behaviors, widespread use of face masks, limitations of travel, and avoidance of activities associated with gathering. All nonessential activities were prohibited until May 2020. Activities were gradually reopened, and free movements within the nation were restored in June 2020. Unfortunately, a new rise in detected Coronavirus disease 2019 (COVID-19) cases was observed: stricter rules were reintroduced in October 2020 to fight the so-called “second” and “third” waves. Respiratory viruses such as influenza, respiratory syncytial virus (RSV), and SARS-CoV-2 have three similar routes of transmission: contact (direct or indirect), droplets, and aerosol transmission. All three viruses replicate in the respiratory tract from where they are subsequently shed and transmit via respiratory secretions [1]. The massive social efforts to prevent the spread of SARS-CoV-2 also affected the spread and the epidemiological features of influenza and RSV. Social distancing played a crucial role in mitigating respiratory infections [2,3]. Furthermore, the preventive efficacy of the vaccination campaign against seasonal flu in the last year must be considered, which involved adults and, for the first time in Italy, all children older than six months, which reduced access to emergency services.

In the last two years, in our tertiary children’s hospital, we observed a significant decrease in the number of children referred to the emergency department [4], partially due to parents’ fear of acquiring COVID-19, who thus managed mild-to-moderate seasonal respiratory illness in their children at home.

Bronchiolitis is a common lower respiratory tract infection that affects neonates and infants, usually from October–November to March–April. This infection is characterized by coryza, persistent cough, and respiratory distress in the presence of wheezing or crackles during chest auscultation. During recent winter seasons (2018–2019), bronchiolitis crowded our hospital wards, often with no beds to manage children with mild-to-moderate respiratory infections. The situation was dramatically changed with the COVID-19 pandemic and related restriction measures, with an evident decrease in the number of cases of bronchiolitis.

This study aims to describe and compare the number of neonates hospitalized for bronchiolitis in the pre-COVID-19 era and the pandemic period in Rome (Italy).

## 2. Materials and Methods

Our internal database was used to attain data for neonates and infants aged up to three months who were admitted to the Medical and Surgical Department of Fetus, Newborn, and Infant in our hospital due to bronchiolitis in the quarters (quarter 1 (Q1)—January–March; quarter 2 (Q2)—April–June; quarter 3 (Q3)—July–September; quarter 4 (Q4)—October–December) of 2018–2020 and the first months of 2021 to compare admissions in the pre-COVID-19 period (January 2018–March 2020) and in the pandemic period (April 2020–February 2022).

In all selected cases, we performed a broad investigation of respiratory viruses by using a multiplex panel capable of simultaneously detecting and identifying 19 clinically relevant virus subtypes: influenza virus (Flu A, Flu A-H1, Flu A-H1pdm09, Flu A-H3, and Flu B), respiratory syncytial virus (A/B), adenovirus, enterovirus, parainfluenza virus (1–4), metapneumovirus, bocavirus, rhinovirus, and human coronaviruses (NL63/229E/OC43). These were detected on nasopharyngeal aspirates (NPA) by multiplex one-step real-time polymerase chain reaction (RT-PCR) using “AllplexTM Respiratory Panel Assays” (Seegene, Korea) on an automated instrumental workflow as previously described [5]. In addition, all NPAs have also been investigated with a multitarget RT-PCR for RNA detection of SARS-CoV-2.

Data are presented as numbers and percentages for categorical variables. Statistical analysis was performed using Microsoft Excel’s software (release 2016 for Windows).

## 3. Results

From 1 January 2018 to 28 February 2022, we admitted 354 infants with bronchiolitis: 243 were admitted in the pre-COVID-19 period and 111 were admitted in the pandemic period. A viral pathogen was found in 342/354 (96.6%) patients. RSV was found to be the virus which was most frequently responsible for diseases (71.2%), followed by rhinovirus (23.6%) and parainfluenza virus (3.9%). Just two cases of bronchiolitis were observed to be caused by SARS-CoV-2; a third patient tested positive for SARS-CoV-2 and RSV simultaneously, but the primary pathogen was considered to be RSV (because of the low viral load of SARS-CoV-2) (Table 1).

**Figure 1:**
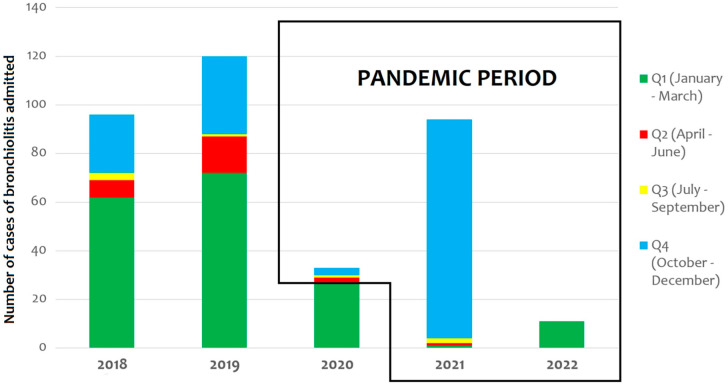
Trends in hospitalizations of neonates and infants due to bronchiolitis over time.

In 2018, 96 infants required hospitalization because of bronchiolitis; during 2019, the number of patients hospitalized increased to 120, while in 2020, only 33 infants were hospitalized (Figure 1).

**Figure 2:**
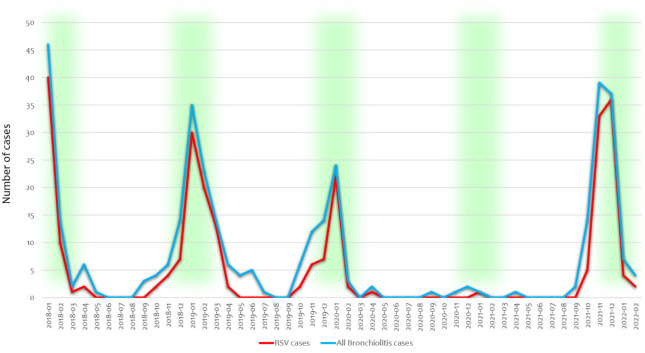
Trends in hospitalizations for all-virus-related bronchiolitis (blue curve) and RSV-related cases (red curve). Green indicates the usual expected bronchiolitis season in Italy before COVID-19, from October–November to March–April, peaking in January–February.

Of these 33 cases, only 6 occurred after the first national Italian lockdown (9 March 2020). In the first quarter of 2021, we observed a similar trend, and only two patients required hospitalization for bronchiolitis (one RSV case, one rhinovirus case). From September 2021, the number of patients with bronchiolitis started to increase (2 infants in the third quarter of 2021, 90 infants in the fourth quarter of 2021, and 11 patients in the first two months of 2022) (Figure 2—blue curve).

RSV infections showed a similar trend. RSV was the leading cause of bronchiolitis both before the pandemic and after the end of social distancing. Among the admitted infants, 66 infections were reported to be caused by RSV (68.8%) in 2018, with 80 RSV infections (66.7%) in 2019 and 25 (75.8%) in 2020. The majority of the latter RSV cases (24/25: 96.0%) occurred before the introduction of lockdown. We hospitalized only 1 infant with RSV in the first nine months of 2021, whereas during October 2021 and February 2022, we admitted 80 infants with RSV (79.2%) (Figure 2—red curve).

RSV was the leading pathogen responsible for bronchiolitis before the national lockdown in March 2020 (70.0% of cases), while rhinovirus was the most prevalent (5/8 patients: 62.5%) during the pandemic when strict restrictions were ongoing (from April 2020 to June 2021) (Table 2). From the beginning of October 2021, RSV returned in prevalence (80/101 cases: 79.2%) among neonates and small infants; furthermore, the epidemiological season started sooner than expected. The reduction in restrictions in Italy in July 2021 increased the number of bronchiolitis due to RSV by 54.2% (from 25% (2 RSV/8 cases) to 79.2% (80 RSV/101 cases)).

## 4. Discussion

For the first time, we accurately report all the pathogens identified to cause bronchiolitis in neonates and infants up to three months of age in the periods before the COVID-19 pandemic and afterwards. Preventive interventions against SARS-CoV-2 implemented in the northern part of the globe have led to a decrease in pediatric, droplet-borne, and contact infections (common cold, gastroenteritis, bronchiolitis, and acute otitis). Conversely, a coincidental reduction in conditions that are not transmitted through droplets and contact, i.e., urinary tract infections, has not been observed [3].

In June 2021, Van Brusselen et al. described bronchiolitis as “a nearly absent disease”, without hiding the fear of a “delayed” peak occurring once most NPIs become more relaxed and pre-pandemic life restarts [6]. Indeed, these measures initially led to a low incidence of influenza and RSV infections in infants and children worldwide [3,6,7,8,9,10]. In France, Rambaud et al. revealed a dramatic reduction in the number of infants admitted to pediatric intensive care units (PICUs) for bronchiolitis during the COVID-19 era, highlighting how this could have a significant impact on infants themselves, infants’ families, and the management of PICU beds [9]. Similarly, in Canada, the annual seasonal epidemics of most seasonal respiratory viruses were observed to be absent in 2020/2021 [11]. Although the impact of each preventive action is challenging to assess, this reduction in hospitalization rates was crucial in countries with health systems close to collapse, such as Brazil [10].

Concerning the Italian situation, after the first national lockdown, the government decided to change its strategy to avoid an economic disaster. It imposed the Traffic Light Approach, a set of stop–start restrictions based on indicators (monitoring capacity, degree of diagnostic capability and contact tracing, transmission dynamics, and resilience of health services) [12]. In this period, we observed a marked decrease in neonatal hospitalizations for bronchiolitis, particularly those caused by RSV, when COVID-19-related restrictions were ongoing. The results we obtained from area-based observations in Rome align with results for Northern Italy and other countries [7,8,9,10,13]. Stera et al. similarly registered a severe drop in hospitalization for bronchiolitis in the city of Bologna, which suggests a severe drop in RSV circulation [14]. Conversely, Curatola et al. described no differences in respiratory viruses’ circulation during the most recent season in a university hospital in Rome; however, excluding the research for RSV, a nasal swab for other respiratory viruses was performed in only 33.1% of the infants included in the study [15]. Furthermore, Vittucci et al. highlighted that the reduction in the circulation respiratory viruses’ could result in a lack of immunity and increased susceptibility to severe infections in following seasons among a cohort of children [16].

Good anti-COVID-19 vaccination coverage has been reached in Italy, and the restrictive measures have been lifted for those with an immunity passport (such as the European Union Digital COVID-19 Certificate—the EUDCC). However, SARS-CoV-2 vaccines do not protect against other respiratory viral infections that have started to spread in a seemingly more aggressive way than in the pre-COVID-19 era. As COVID-19 vaccines became more publicly accessible, the Italian government extended the requirement of the EUDCC, also known as a “Green Pass”, to attend sports events and concerts and for access to indoor places, such as bars, restaurants, and gyms, as well as for travel. Meanwhile, cases of bronchiolitis were reported to rise globally.

In Japan, the comparison of 2021 weekly RSV activity with activity in 2017–2020 from the Tokyo Metropolitan Infectious Disease Surveillance Center demonstrated the most significant annual increase in cases for 2021 [17]. An RSV surge has been similarly reported in Australia as social distancing restrictions were relaxed. The authors hypothesized that the expanded cohort of RSV-naïve patients, including an increased number of older children and waning population immunity [18], may have contributed to this marked resurgence [19]. A reemergence of RSV bronchiolitis has been recently observed in Wales at a rapid rate that is out of sync with the usual seasonal pattern [20]. According to our data in Italy, RSV returned to prevalence from the beginning of October 2021, with a reduction in prevalence compared to the pre-pandemic era; this reduction was probably due to the mandatory masks indoors in Italy and the increased awareness of the need for hand washing.

Although the association between SARS-CoV-2 and bronchiolitis has been reported [21], we found only two cases of bronchiolitis due to SARS-CoV-2 in our hospital. Considering the fact that both term and preterm newborns appeared to have a lower expression of SARS-CoV-2 entry receptors in their nasal epithelium than adults [22], and that infants seem to experience a milder course [23], a limitation of our study could be the exclusion of infants who were not hospitalized.

Hospitalization in bronchiolitis usually only occurs with the presence of hypoxia, moderate–severe respiratory distress, dehydration, or apnea; other criteria to be considered are comorbidities (such as prematurity, bronchopulmonary dysplasia, or congenital heart diseases) and unfavorable social and environmental factors [24]. Therefore, an accurate estimate of respiratory viruses’ circulation is difficult to perform, excluding patients managed at home.

Recently, Camporesi et al. explored the epidemiology, microbiology, and severity of bronchiolitis in four pediatric referral centers located in different geographical areas in Italy during 2021–2022 [25]. Their results were similar to ours; they detected the first cases during the summer of 2021, which peaked in November 2021 and declined into December 2021, with only a few cases detected in January 2022. This confirms that the 2021–2022 bronchiolitis season in Italy started and peaked earlier than in the pre-pandemic seasons. However, they included all children younger than two years of age. Conversely, we precisely reported the identified pathogens causing bronchiolitis in neonates and small infants before the COVID-19 pandemic and afterwards. These data could be helpful in planning specific preventive measures for neonates and small infants against future bronchiolitis “waves” that can present earlier than expected, as we learned, due to changes in restrictive measures and the viral interference between respiratory viruses [26].

## 5. Conclusions

We observed a dramatic drop in admissions of infants with bronchiolitis due to RSV with the implementation of SARS-CoV-2-related social restrictions. We noted fluctuations were related to the intensity of restrictive measures at a given time: we learned that bronchiolitis in neonates and small infants could be drastically contained when non-pharmaceutical interventions hinder transmission from adults and older children. Indeed, since the restrictions were relaxed, cases have started to rise again with a significant trend.

A lesson learned during lockdowns tells us that simple preventive measures should not be forgotten, because they can markedly reduce bronchiolitis-related hospitalization of neonates and infants. This is especially the case among those younger than three months or with pre-existing risk factors (such as prematurity, bronchopulmonary dysplasia, or congenital heart diseases) who thus have a higher risk of severe illness and hospitalization which goes beyond the protection provided by RSV prophylaxis.

## Figures and Tables

**Table 1:** Identified microorganisms causing bronchiolitis in neonates and infants up to three months of age.

	2018		2019		2020		2021		2022	
	RSV	51	RSV	63	RSV	24	RSV	1	RSV	6
	Rhinovirus	5	Rhinovirus	6	Rhinovirus	1			Rhinovirus	2
	Influenza	1	Influenza	1	Coronavirus *	2			SARS-CoV-2	2
	Bocavirus	1	Bordetella pert.	1					Metapneumov.	1
	Not identified	4	Not identified	1						
**Q1**	Total	62	Total	72	Total	27	Total	1	Total	11
	RSV	2	RSV	2	RSV	1	Rhinovirus	1		
	Rhinovirus	3	Rhinovirus	8	Not identified	1				
	Parainfluenza	1	Parainfluenza	2						
	Adenovirus	1	Adenovirus	1						
			Not identified	2						
**Q2**	Total	7	Total	15	Total	2	Total	1		
	Rhinovirus	2	Coronavirus **	1	Rhinovirus	1	Rhinovirus	2		
	Not identified	1								
**Q3**	Total	3	Total	1	Total	1	Total	2		
	RSV	13	RSV	15	Rhinovirus	3	RSV	74		
	Rhinovirus	7	Rhinovirus	12			Rhinovirus	7		
	Parainfluenza	2	Parainfluenza	2			Parainfluenza	3		
	Coronavirus ***	1	Adenovirus	1			Bocavirus	1		
	Not identified	1	Not identified	2			Metapneumov.	5		
**Q4**	Total	24	Total	32	Total	3	Total	90		
***Total***		***96***		***120***		***33***		***94***		***11***

Among coronavirus cases: * 1 OC43 and 1 HKU1. ** 1 229E. *** 1 OC43.

**Table 2:** Timeline of the COVID-19-pandemic-related measures in Italy.

Period	Date	Measure
**Pre-pandemic period**	Before February 2020	No restrictive measures for Italian citizens
	22 February 2020	Quarantine from 11 municipalities in Northern Italy (the “Red Zones”)
	8 March 2020	Expansion of the quarantine to all of Lombardy and 14 northern provinces
	9 March 2020	Order of national lockdown in the whole country
**Pandemic** **period**	3 June 2020	End of national lockdown
	7 October 2020	New imposition of the use of protective masks outdoors
	25 October 2020	New restrictions with a “traffic light” color code (red, orange, and yellow zones), imposing the online schooling for 75% of secondary schools and university students, the closing of gyms, swimming pools, theaters, and cinemas, as well as the closing of bars and restaurants before 6 p.m. based on the number of infections
	28 June 2021	Slowdown of restrictive measures, with the introduction of the “Green Pass” for vaccinated or recovered subjects or those with a proof of a negative test taken within the last 48 h; no requirement to wear masks outdoors
	1 September 2021	Full reopening of secondary schools and universities for in-person teaching
	8 October 2021	Full reopening of theaters, cinemas, and concert halls
	6 December 2021	Introduction of a two-tiered Green Pass system, named the “Super Green Pass” to access events, venues, and services and to travel on all local and long-distance public transport
	7 January 2022	Introduction of the vaccination obligation for people aged 50 or over, with a penalty for non-compliant persons

## Data Availability

All relevant data are presented in the study. All data generated or analyzed during this study are included in this published article. Further inquiries can be di-rected to the corresponding author.
